# The relationship between work stress and work ability among power supply workers in Guangdong, China: a cross-sectional study

**DOI:** 10.1186/s12889-016-2800-z

**Published:** 2016-02-06

**Authors:** Hualiang Li, Zhiting Liu, Runzhong Liu, Li Li, Aihua Lin

**Affiliations:** 1 Electric Power Research Institute of Guangdong Power Grid Corporation, Guangzhou, 510080 PR China; 2 Department of Medical Statistics and Epidemiology, School of Public Health, Sun Yat-sen University, No. 74, Zhongshan 2nd Road, Guangzhou, 510080 PR China

**Keywords:** Power supply workers, Work stress, Work ability, Structural equation model

## Abstract

**Background:**

Faced with the challenge of population aging, a prolonged working life is increasingly important in today’s society. Maintaining work ability of employees is one of the effective ways to cope with the challenges to sustainability of the workforce presented by population aging. Researchers have shown ongoing interest in exploring the determinants of restricted work ability. The aim of this study was to evaluate the effects of work stress on work ability among power supply workers in Guangdong, China.

**Methods:**

A cross-sectional study was conducted among power supply workers during August 2014 to September 2014. A total of 805 subjects were enrolled in the study. Work stress was assessed by the Job Content Questionnaire and the Effort Reward Imbalance Questionnaire. Work ability was assessed by the Work Ability Index (WAI). The structural equation model was applied to test the relationship between different work stress components and work ability simultaneously using the Job Demands-Resources model as a framework.

**Results:**

Job resources (measured by job control, reward and social support) were positively and directly associated with work ability (*β* = 0.70, *P* < 0.001). The association between job demands and work ability was also statistically significant (*β* = −0.09, *P* = 0.030). In addition, the findings also supported previous studies in that job demands were correlated with job resources (*β* = −0.26, *P* < 0.001).

**Conclusions:**

Our findings suggest that decision makers and health care providers should consider increasing job resources available to power supply workers. Consideration of organizational changes related to the design of the job task also would be useful to improve the employees’ work ability.

## Background

Most western countries are experiencing the challenge of population ageing. Demographic projections of 27 European countries by Eurostat (2011) indicate that the old-age dependency ratio –the ratio of those outside the labor force to those of working age (15-64y)- will double from 25.9 % on average in 2010 to 50.2 % by 2050 [1]. The demographic projections in China are not different, with the population aged 65 and older expected to be almost 300 million by 2050 [2]. With the rapidly ageing population, it is of utmost importance to prevent disability and early retirement so that everybody can remain in the labor market for as long as possible. Promoting work ability is one of the effective ways to cope with the challenges to sustainability of workforce presented by population ageing. Prospective studies have demonstrated that a low score of work ability index (WAI), which is an instrument used in clinical occupational health and research to assess work ability, increased the risk of premature work exit due to disability pension or early retirement [3, 4].

Previous studies revealed that work ability was not only associated with individual characteristics but also with work-related factors [5, 6]. Epidemiologic studies have shown that work stress is related to work ability [7, 8]. The Job Content Questionnaire (JCQ) and Effort Reward Imbalance (ERI) Questionnaire have been widely used to assess work stress. The Job Content Questionnaire, based on the Job Demand-Control-Support model, demonstrates that employees who have high job demands while simultaneously having little control of their work are in a high job-strain situation. In addition, this model argues that work stress increases when the level of social support decreases. Previous studies showed that decreases of work ability were related to high job strain due to high demand and low control [8]. The Effort Reward Imbalance Questionnaire based on Effort Reward Imbalance model states that the experience of a lack of reciprocity in terms of high ‘cost’ and low ‘gains’ elicits negative emotions. The negative association between ERI and work ability have been supported by both cross-sectional and longitudinal studies [9, 10].

As a consequence of rapid economic development, domestic consumption of electricity in China has increased rapidly during the past decades. The total electricity consumption in China during 2014 was the highest in the world and accounted for 57 % of the consumption in Asia (https://yearbook.enerdata.net/). Chinese workers in the power supply industry have experienced increasing workload. As a result of the commercialization of the power industry in China, the labor market has undergone significant changes in the past several years. Deregulation, privatization and reduction of welfare programs have resulted in less job security. In addition, workers employed in the power supply industry are exposed to multiple stressors including electric shock, injury and trauma resulting from accidents, poor body posture, bad weather conditions, outdoor work and shift work [11]. Long-term stay in isolated regions also increases the level of work stress. It is of utmost importance to explore the relationship between work stress and work ability among power supply workers in China.

Most of the aforementioned studies concerning the relationship between work stress and work ability were conducted in western societies. However, work conditions in China are different from the ones in western societies. Long hours, rough conditions, and low pay have resulted in increased work stress. Besides, the Chinese workers are hardworking and obedient due to the culture of nationalism, stability, and harmony. This combination has created a uniquely motivated population. It is unclear whether the relationship between work stress and work ability in western societies could be generalized to China. In addition, despite that there were several studies explored the relationship between work stress and work ability in China [12, 13], few studies have applied a theoretical model to test the associations. The risk factors of work stress are correlated and individuals do not experience them in isolation. It is more appropriate to analyze effects of all these factors simultaneously by a theoretical model.

The job demands-resources (JD-R) model provides an opportunity for us to understand the work ability by a theoretical model [14, 15]. The basic premise of the JD-R model is that whereas every occupation may have its own specific risk factors associated with job stress, these factors can be classified into two general categories (i.e., job demands and job resources), thus constituting an overarching model that may be applied to various occupational settings, irrespective of the particular demands and resources involved [14, 15]. Evidence for this hypothesis would offer organizations a tool to maintain work ability by optimizing job demands and increasing job resources.

According to the JD-R model, job demands refer to those physical, social or organizational aspects of the job that require sustained physical or mental effort and are therefore associated with certain physiological and psychological costs [15]. Demands of the job have been linked to work ability in previous cross-sectional studies [6, 7, 16]. Both the mental and physical job demands are seen as having a negative relationship with work ability.

Job resources are defined as “those physical, psychological, social, or organizational aspects of the job that may be functional in achieving work goals, reduce job demands and the associated physical and psychological costs, and stimulate personal growth or development” [15]. Previous studies indicated that job resources were related to intrinsic motivational processes through fostering skill development and growth. In addition, they are instrumental in helping employees achieve work goals [17]. Therefore, some studies have examined the relationship between job resources and work ability [16]. Besides, according to the definition of job resources and previous study job resources may be correlated with job demands.

Consequently, we formulated three hypotheses in the present study. Firstly, it was supposed that job demands would be negatively related to perceived work ability. In addition, job resources would be positively related to perceived work ability. Thirdly, it was supposed that job demands would be correlated with job resources.

The aim of this study was to explore the relationship between work stress and work ability among Chinese power supply workers using a structural equation model. The findings of this study may help devise new strategies to improve ability.

## Methods

### Setting and participants

This cross-sectional study was conducted in a power supply company in Guangdong Province, China. The company supplies power for 24 townships, with 1800 square kilometers service area. By August 2014, the company employed 3094 workers, and consisted of 24 branches, each with a similar organizational structure.

To facilitate data collection, a cluster sampling method was applied to select the participants. Eight branches were randomly selected from the company. The questionnaire was related to work characteristics, and thus limited to subjects currently working. Retired employees and those on a long-term leave were excluded. In total, 924 workers took part in the survey. The response rates for each of the 8 selected branches were 56.2 %, 42.5 %, 55.3 %, 44.8 %, 51.9 %, 44.9 %, 85.5 %, and 77.3 %, respectively. Subjects with missing data were excluded from the analysis, resulting in a study sample of 805 individuals.

### Ethics statement

This study was approved by the Institutional Review Board of the School of Public Health, Sun Yat-sen University. All participants gave written informed consent prior to administering the survey.

### Data collection

The data collection was carried out from August until September 2014, using an anonymous self-administered questionnaire. The questionnaire included socio-demographic characteristics (gender, age, education level, marital status, monthly income, shift work and exposure to occupational hazards), and questions related work stress and work ability.

### Work stress

Work stress was assessed by two validated Chinese version questionnaires [18–20]: Job Content Questionnaire (JCQ) and Effort Reward Imbalance-Questionnaire (ERI Questionnaire).

The 22-items JCQ, which is based on the JDCS model, consists of 3 scales [18], psychological job demands (5 items), job control including skill discretion (6 items) and decision authority (3 items), and social support including coworker support (4 items) and supervisor support (4 items). Four-point Likert-type scales from “strongly disagree” to strongly agree” were utilized to answer all these items. Responses were combined into summary scales, where higher scores indicate greater demand, control or support. The job strain variable was constructed by dichotomizing the scale scores at the median of the sample distribution and combined them into one variable. The job strain was defined as the combination of high demands and low control.

The 17-items ERI questionnaire consists of 2 domains, termed effort (6 items) and reward (11 items, including the financial reward, esteem reward, and career opportunities) [21]. Participants responded to the items of effort in two steps. If they agreed with the statements in the first step, they were asked to evaluate to what extent they usually feel distressed by this typical experience. The rating procedure was defined as follows with higher ratings pointing to higher efforts: (1) does not apply; (2) does apply, but subject does not consider herself or himself distressed; (3) does apply and subject considers herself or himself somewhat distressed; (4) does apply and subject considers herself or himself distressed; (5) does apply and subject considers herself or himself very distressed. The rating procedure of reward was similar to effort. After variable recoding procedures, lower ratings were equivalent to lower rewards. The ratio between the two scales ‘effort’ and ‘reward’ is calculated to quantify the degree of mismatch between high cost and low gain. According to the established recommendations an ER ratio > 1 implies an effort reward imbalance [21].

The JCQ is focused on work overload and time pressure as indicators of job demands, on skill discretion and decision latitude as indicators of job control, and on supervisor support and coworker support as indicators of social support [22]. The ERI questionnaire emphasizes the effort (extrinsic job demands and intrinsic motivations to meet these demands) and the reward (in terms of salary, esteem, and career opportunities) [17, 21]. Even though the JDCS and ERI models may overlap partially in the dimensions of psychological job demands and effort, they have complementary dimensions (e.g. job control, social support and reward) which could broaden the psychosocial scope [23]. A previous study indicated that the subcomponents of JCQ and ERI may capture different aspects of workplace stress, and each of these subcomponents has the potential to measure the psychosocial factors in workplace [24]. In this study, job resources were indicated by two JDCS subscales (job control, social support) and one ERI domain (reward). Because the subscales of psychological job demands and effort may overlap, we chose one of them as the job demands dimension according to the fit indices of confirmatory factor analysis.

### Work ability

Work ability was measured by the Chinese version of the Work Ability Index (WAI) [25, 26]. The WAI consists of 7-parts items: (1) current work ability compared with the lifetime best, (2) work ability in relation to the demands of the job, (3) number of current diseases diagnosed by a physician, (4) estimated work impairment due to diseases, (5) sick leave during the past year, (6) own prognosis of work ability 2 years from now and (7) mental resources. According to previous studies [27], we combined the 7 items into 3 domains based on the purpose of WAI: 1) perceived work ability, including items 1, 2 and 6 of WAI; 2) worker’s health status, including items 3, 4 and 5; and 3) mental resources, including item 7.

### Control variables

These included three variables – age, shift work and exposure to occupational hazards. Age was continuous variable, and shift work (yes = 1/no = 0) and exposure to occupational hazards were dichotomous variables (yes = 1/no = 0). Exposure to occupational hazards was assessed based on a self-reported measure, and was defined as contacting with physical or chemical hazards, such as noise, electric shock, bad weather conditions and chemical poisons. Control variables were included in the model, with direct associations with work ability [28].

### Statistical analysis

First, descriptive statistics were calculated to demonstrate socio-demographic characteristics of participants and major study variables, such as psychological job demands, job control, social support, reward, effort, perceived work ability, health status and mental resources. The Little’s MCAR test was performed to assess the pattern of missing data. As the test was not significant at the level of 0.05, we concluded that missing data occurred at random and the deletion of individuals with missing data was presumed to result in a random subsample of the original target sample. The major study variables were assessed to ensure normality by evaluating skewness and kurtosis. For the skewness index, absolute values greater than 3.0 are extreme. Kurtosis is an index of the peak and tails of the distribution. Absolute values higher than 10.0 for the kurtosis index suggest a problem, and values higher than 20.0 are extreme [29]. The skewness of study variables ranged from 0.02 to 1.13, and kurtosis statistics ranged from 0.18 to 2.97 (Table 2). Therefore, no attempts (e.g. transformation) were made to improve the distributional properties.

The next step was to analyze the correlation matrix of major observed variables of latent constructs.

Lastly, the structural equation model (SEM) was employed to establish a comprehensive assessment of the relationship between work stress and work ability. A two-step analytic approach was employed by first estimating measurement models using confirmatory factor analysis (CFA) and then testing a structural model. The analysis was assessed with the maximum likelihood (ML) estimator. Overall, data from 805 workers were included in the study. Schreiber [30] suggests that a sample size should have at least 20 cases for each parameter for SEM. In this study, there were 22 free parameters, indicating adequate power from the sample size. Absolute and comparative fit indices were used to evaluate whether the data fit the hypothesized model. Because the Chi-square statistic is affected by the sample and model size, the following fit indices were considered at the same time. A root mean squared error of approximation (RMSEA) <0.08, standardized root mean square residual (SRMR) <0.05, a goodness of fit index (GFI), comparative fit index (CFI), and Tucker-Lewis index (TLI) of 0.90 or above indicated an acceptable model fit [31]. Data were analyzed using SPSS (Chicago, IL, USA) version 20.0 and AMOS (Chicago, IL, USA) version 18.0 software.

## Results

### Descriptive analysis

This study comprised 805 workers (73.9 % males). The mean age of the participants was 35.22 (SD = 8.15) years, of whom 66.2 % had a university degree. About 30.4 % were administrators and 69.6 % were front line workers. The front line workers included substation operators, maintenance personnel, electrical test personnel, power system control operators and workers in electric transmission line. Overall, high job strain was experienced by 28.7 % participants, and 19.4 % of the employees reported an ER ratio >1. Other socio-demographic characteristics are shown in Table 1.

**Table 1:** Demographic characteristics of participants (*n* = 805)

Demographic characteristics	Number	Proportion (%)
*Gender*		
Male	595	73.9
Female	210	26.1
*Age group, year*		
<30	247	30.7
30~	323	40.1
40~	192	23.9
50~	43	5.3
*Education level*		
High school/technical secondary school or less	105	13.0
Junior college	167	20.8
University or above	533	66.2
*Personal monthly income*		
<¥4000	210	26.1
¥4000~	224	27.8
¥6000~	270	33.5
¥8000~	101	12.6
*Marital status*		
Single	192	23.9
Married	602	74.8
Divorced/widowed	11	1.4
*Shift work*		
Yes	78	9.7
No	727	90.3
*Exposure to occupational hazards*		
Yes	699	86.8
No	106	13.2
*Work role*		
Administrators	245	30.4
Front line workers	560	69.6
*Job strain*		
Yes	231	28.7
No	574	71.3
*ERI*		
Yes	156	19.4
No	649	80.6

The correlation matrix, means and standard deviations of the observed indicators are displayed in Table 2. The correlations were generally in the expected directions. Most indicators of job demands were negatively and significantly related to job resources indicators (5 out 6 correlations), and work ability indicators (all the correlations). All the indicators of job resources were positively and significantly associated with work ability indicators.

**Table 2:** Means, scale range, standard deviations and correlations among observed variables of latent constructs

	1	2	3	4	5	6	7	8	9
*Job demands*									
1. psychological job demands									
2. effort	0.49^**^								
*Job resources*									
3. job control	−0.12^**^	−0.19^**^							
4. social support	−0.06	−0.25^**^	0.39^**^						
5. reward	−0.25^**^	−0.53^**^	0.39^**^	0.42^**^					
*Work ability*									
6. mental health	−0.19^**^	−0.38^**^	0.35^**^	0.34^**^	0.45^**^				
7. perceived work ability	−0.09*	−0.20^**^	0.25^**^	0.17^**^	0.22^**^	0.43^**^			
8. health status	−0.23^**^	−0.37^**^	0.21^**^	0.20^**^	0.32^**^	0.43^**^	0.39^**^		
*Control variable*									
9. age	−0.01	0.03	0.03	−0.14**	−0.02	0.16**	−0.02	−0.03	
Mean	32.89	16.81	61.20	23.90	44.81	2.97	15.75	21.91	35.22
Scale range	12 ~ 48	6 ~ 30	24 ~ 96	8 ~ 32	11 ~ 55	1 ~ 4	3 ~ 27	3 ~ 18	21 ~ 60
Standard deviation	4.29	5.43	8.23	3.22	8.39	0.82	2.15	3.26	8.15
Skewness	0.72	−0.02	−0.32	0.03	−0.82	−0.30	−0.58	−1.13	0.55
Kurtosis	0.86	−0.45	0.78	2.97	0.18	−0.67	1.39	0.83	−0.42

*Notes:* ^*^ *P* < 0.05; ^**^ *P* < 0.01

### Measurement models

The first-order confirmatory factor analysis was estimated in 3 latent variables (job demands, job resources and work ability) in the hypothesized model. If the job demands dimension was designated as effort, the measurement model did not fit the data adequately (χ^2^_(12, N=805)_ =116.546, GFI = 0.958, CFI = 0.920, TLI = 0.859, RMSEA = 0.104, SRMR = 0.052). We then chose the JCQ subscale of psychological job demands as the job demands dimension. This model proved a close fit to the data (χ^2^_(12, N=805)_ =67.268, GFI = 0.977, CFI = 0.947, TLI = 0.908, RMSEA = 0.076, SRMR = 0.040), providing evidence to not reject the dimensional structure proposed. The difference of chi-square between these two models was 49.278. Consequently, the subscale of psychological job demands was chosen as the dimension of job demands.

The composite reliability (CR) was used to measure the internal consistency for each of the latent variables. According to Fornell and Larcker, CR should be above 0.60 [32]. In this study, CR coefficients for job resources and work ability were 0.66 and 0.68, respectively. In terms of validity, all factor loadings between each latent variable and its indicators were statistically significant at the 0.001 level, indicating that the indicators of job demands, job resources and work ability, were valid. These results confirmed the factor structure in the model.

### Structural model

**Fig. 1:**
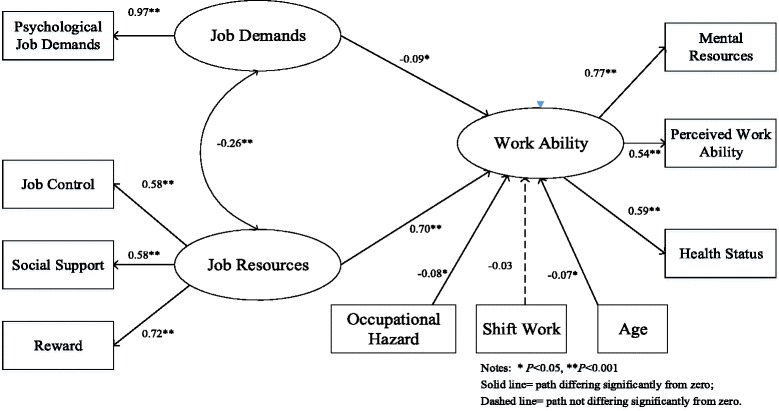
Structural model with standardized path loadings

Next, the control variables were added to the model as an observed variable. A structural model was established to assess the relationship between work stress and work ability. The model indicated a Chi-square of 174.382 (degree of freedom = 33, *N* = 805), GFI of 0.960, CFI of 0.876, TLI of 0.831, RMSEA of 0.073 and SRMR of 0.056, suggesting an acceptable model fit (Fig. 1).

*Hypothesis 1: Job demands are negatively related to perceived work ability.*

The first hypothesis was supported by the structural model (Fig. 1). Job demands were negatively and significantly associated with work ability (*β* = −0.09, *P* = 0.030).

*Hypothesis 2: Job resources are positively related to the perceived work ability.*

The second hypothesis was also proved. Job resources were significantly and positively associated with work ability (*β* = 0.70, *P* < 0.001), indicating that workers with higher job resources would have a better work ability.

*Hypothesis 3: Job demands are correlated with job resources.*

In our study, job demands had a negative and significant relationship with job resources (*β* = −0.26, *P* < 0.001), showing that workers experiencing higher job demands report poorer job resources.

Additionally, self-reported exposure to occupational hazards was significantly and negatively associated with work ability (*β* = −0.08, *P* = 0.026), suggesting that employees exposed to occupational hazards might be at the risk of decreasing work ability. In our study, age was related to work ability (*β* = −0.03, *P* = 0.046). Doing shift work was not associated with work ability.

## Discussion

This study was designed to explore the relationship between work stress and work ability among Chinese power supply workers using SEM. The results supported the tested model that work ability was associated with job demands and job resources. In addition, the negative association between job demands and job resources was also statistically significant.

The negative relationship between work stress and WAI has been found in diverse occupational groups [7–10, 33, 34]. The JCQ developed by Karasek and the ERI questionnaire developed by Siegrist are two common instruments to measure work stress. However, few studies have applied a structural equation model to the combined analysis of all the components (psychological job demands, job control, social support, reward) in the Job Content Questionnaire and Effort Reward Imbalance Questionnaire.

The JD-R model was used to test the association between work stress and work ability in this study. The JD-R model, which has been previously applied to understand work ability [16], provides theoretical grounding to a construct that has been almost exclusively examined without a theoretical framework. According to the structural model in this study, we observed a positive association between job resources and work ability. This is consistent with previous studies that reported more job resources may directly increase work ability [35]. Our hypothesis regarding job demands and work ability was also supported by the data. Several studies reported a negative and significant association between job demands and work ability [7, 36]. McGonagle examined multiple job demands and resources relating to perceived work ability in health care workers from six nations [16]. In their study, the job demands were identified as four specific types: role demands, work environmental demands, emotional demands, and physical demands. They tested effects of all demands and resources simultaneously and found the association between role demands with work ability in an Australian sample reached statistical significance. A systematic review revealed that both the psychosocial and physical job demands were associated with work ability [6].

Age was incorporated in the model as a control variable. In our study, age was negatively associated with work ability. This was consistent with several previous studies [37, 38]. However, a previous study proposed that if health was considered, age did not play an important role in work ability [16]. Aging and work ability are currently one of the great challenges for research in this field. Longitudinal and well-designed studies are warranted to clarify this question. As expected, exposure to occupational hazards was related to work ability. Workers employed in the power supply industry are exposed to multiple stressors, such as injury and trauma resulting from accidents, poor body posture, bad weather conditions and outdoor work. A previous systematic review indicated that thermal discomfort and poor physical climate were related with a lower WAI [6].

We found a negative relationship between job demands and job resources. This is in accordance with an Australian study, indicating that higher job demands are related to lower job resources [39]. Given that job demands and job resources were measured concurrently, the relationship between these two variables is not necessarily causative.

This study contained a relatively large sample size. In addition, the structural equation model used in this study allowed simultaneous evaluation of multiple relationships between different work stress components and work ability. However, there are several limitations in our study. Firstly, the cross-sectional design by which exposure and outcome were measured concurrently does not permit causative conclusions. In epidemiological studies, an association of exposure and outcomes is causal only if the exposure precedes outcomes. Secondly, self-reported measurements of work stress and work ability may lead to recall bias. Also, exposure to occupational hazards was assessed by a self-reported measure, and information bias are still possible. Worker’s awareness of the occupational hazards could affect the measure’s correctness. However, the department of work safety supervision in this company had provided a lot of the health education lessons to the employees. The contents of the lessons included the introduction of the occupational hazards in the workplace and the way to protect themselves. Workers in the company were familiar with their work conditions, and they were able to recognize the occupational hazards in their workplace. Thirdly, considering that the average age of the study population was 35 years and more than 70 % were younger than 40 years, the variance of age was limited for us to explain the relationship between age and work ability. Despite these considerations, the profile of the study sample is similar to the target population (employees in this company) in terms of age, and it reflects the characteristics of the power supply workers in this company. Other well-designed studies are warranted to clarify this question. Lastly, the power supply workers may not be representative of workers elsewhere in different work settings. Generalizations beyond the study population should be made with caution.

The findings have several practical implications for organizations and individuals. In order to improve the work ability, policy makers could focus on optimal job demands and sufficient job resources. To optimizing job demands, policy makers should adopt several organizational changes concerning the design of the job task. The policy makers might consider increasing job resources by improving job control, providing professional development opportunities and supervisor support, and facilitating co-workers interaction. Workplace health promotion could also focus more on improving human relationships by training of leadership skills, conflicts resolutions, and communication skills.

## Conclusions

In conclusion, this study determined the relationship between work stressors and work ability among power supply workers. According to the results, resources and demands were related to work ability. It is suggested that increasing job resources may be an important strategy to improve the work ability among power supply workers. The decision makers of organizational policies might consider increasing the job resources by improving worker’s job control, providing adequate supervisor support, facilitating co-worker interaction and providing reasonable rewards (e.g. salary, esteem, job promotion and job security). In addition, several organizational changes concerning the design of the job task should be considered to improve work ability. Furthermore, longitudinal studies are needed to better identify the association between work stress and work ability in similar and other occupational settings.

## Abbreviations

JDCS model: Job demand-control-support model
ERI model: Effort reward imbalance model
JD-R model: Job demands-resources model
JCQ: Job content questionnaire
WAI: Work ability index
RMSEA: Root mean squared error of approximation
SRMR: Standardized root mean square residual
GFI: Goodness of fit index
CFI: Comparative fit index
TLI: Tucker-Lewis index
